# Foliar application of *Halocnemum strobilaceum* improves *Chenopodium quinoa* growth and physiological traits for saline agricultural residence

**DOI:** 10.3389/fpls.2025.1688246

**Published:** 2025-11-25

**Authors:** Ghalia S. Aljeddani, Amal M. Fakhry, Ameina S. Almoshadak, Soliman M. Toto

**Affiliations:** 1 Department of Environmental Sciences, College of Science, University of Jeddah, Jeddah, Saudi Arabia; 2 Department of Botany and Microbiology, Faculty of Science, Alexandria University, Alexandria, Egypt; 3 Department of Biological Sciences, Faculty of Science, King Abdulaziz University, Jeddah, Saudi Arabia

**Keywords:** sustainable agriculture, *Halocnemum strobilaceum*, arid land, biostimulant, eco-friendly salinity management, NaCl-induced abiotic stress, foliar application under salinity, salt stress mitigation in crops

## Abstract

Salinity is a major abiotic stress limiting crop productivity, particularly in arid and semi-arid regions. This study evaluated the effectiveness of *Halocnemum strobilaceum* extract (HE) as a foliar biostimulant to improve growth, yield-related traits, and physiological performance of *Chenopodium quinoa* under controlled saline conditions (0–150 mM NaCl), representing productivity-limiting but non-severe salinity levels. Elevated salinity significantly reduced root length (- 17.4%), leaf area (- 44.3%), and seed weight (- 26.4%). HE application mitigated these effects, enhancing leaf area (+8.6%) and the weight of 1000 seeds (+33.9%) under moderate to high salinity. Physiological analysis revealed that HE increased photosynthetic efficiency (Fv/Fo) with improvements of 44.3% at 100 mM and 12.4% at 150 mM NaCl., reduced oxidative damage by lowering H_2_O_2_ (up to −32.7%) and malondialdehyde (−39.2%) levels, and increased protein (+25.4%) and lipid content (+24.2% under 0 and by 16.5% under 50 mM NaCl). SDS-PAGE revealed salinity-induced changes in quinoa seed proteins, with loss of specific bands and appearance of two novel bands (43, 30 kDa) in the protein profile of HE-treated salinized quinoa plants under 150 mM NaCl. HE application is associated with improvement in physiological traits and yield components by promoting osmotic adjustment, maintaining membrane integrity, and enhancing antioxidant defenses. The SDS-PAGE results show qualitative changes in the protein profile, highlighting HE's role in modulating quinoa's proteomic response to saline conditions.

## 1 Introduction

Salinity is one of the major environmental constraints that has negative effects on plant growth and development. Globally, approximately 800 million hectares of the Earth’s land are salt-affected, accounting for around 6% of the total land area, and this is expected to increase in the near future (Abdel Latef et al., 2020; Ruiz-Lozano et al., 2012). The consequences of soil salinity as a major environmental problem are evident in arid and semi-arid regions worldwide (Ahmad et al., 2015; Liu et al., 2016; Osman et al., 2021). Plants grown in salty environments tend to accumulate high levels of salt, which cause disturbances within the physiological and biochemical parameters, leading to ion toxicity, reduced photosynthesis, free radicals’ accumulation, instability of membranes, and a variety of metabolic disorders (Atzori et al., 2017; Hanin et al., 2016; Osman et al., 2021). The damage triggered by salinity is influenced by many factors, of which salt concentration, crop sensitivity, and management control tools are the most important (Carillo et al., 2019; IUCN Red List categories and criteria: Version 3.1 (2nd ed.). Gland, Switzerland, and Cambridge, UK: IUCN; Rouphael et al., 2012). The adaptive strategy of salt-tolerant plants in response to salinity stress takes place by different mechanisms, including osmoregulation, antioxidant defense system, and toxic ion (Abdel Latef et al., 2020). Therefore, studying plant mechanisms under salinity stress is essential to determine whether plants can perform physiological and biochemical processes to cope with salinity stress. Furthermore, the use of sustainable agricultural tools to enhance plant growth and productivity under salt stress can ensure the establishment of high-quality crops under anticipated climate change. It is important to identify environmentally safe and sustainable approaches to mitigate the harmful effects of salinity on plants. One of the strategies to mitigate salt stress is the use of natural plant extracts instead of chemical solutions, for limiting soil, water, and environmental pollution. Natural plant extracts are effective in regulating metabolism, thereby promoting plant growth and yield.

Halophytes distributed along the Red Sea and the Mediterranean Sea coastal lands could be used as a valuable source of bio-stimulants for mitigating salinity stress (Osman et al., 2021). Among them, *Halocnemum strobilaceum* (Pall.) M. Bieb is of particular interest due to its remarkable salt tolerance and adaptive mechanisms that allow it to survive and reproduce in highly saline environments. This small succulent shrub belonging to the family Amaranthaceae is capable of germinating at salinity levels up to 0.5 M NaCl, (Qu et al., 2008) reflecting its efficient osmoprotective and ion-regulation systems. Furthermore, *H. strobilaceum* is known to possess strong antioxidant potential, with its ethyl acetate extract and flavonoid components exhibiting high radical-scavenging activity comparable to that of standard antioxidants such as Trolox (Radwan and Shams, 2007). The plant’s volatile oil is also rich in hydrocarbons and oxygenated compounds, suggesting potential bioactivity. Owing to these characteristics, *H. strobilaceum* represents a promising candidate for developing eco-friendly bio-stimulants aimed at enhancing the resilience of salt-sensitive crops like quinoa.

Quinoa (*Chenopodium quinoa* Willd.) is one of the most important economic plants, belonging to the Amaranthaceae family. Its native range is Ecuador to northwestern Argentina, and it is introduced to many countries across the globe. Quinoa grains are highly healthful due to their high protein content and numerous vitamins and minerals. It is also used as a medicinal plant, treating some medical disorders. Quinoa’s ability to produce high-protein grains under ecologically extreme conditions makes it important for the variations of future agricultural systems, especially in saline areas of arid lands (Bhargava et al., 2006). Therefore, the main aim of our study is to evaluate the potential cultivation of quinoa plants in saline soils and to assess the effect of *H. strobilaceum* extract as a bio-stimulant on growth and yield-related traits of quinoa under saline conditions by estimating growth traits, physiological attributes, some enzymatic activities, and protein patterns in salinized quinoa plants. To the best of our knowledge, no study has evaluated the foliar application of *H. strobilaceum* extract on quinoa under salt stress. The present study explores the potential of a naturally salt-tolerant halophyte as a sustainable source of bioactive compounds that may support quinoa performance under saline conditions.

## 2 Materials and methods

### 2.1 Halocnemum strobilaceum collection and extract preparation

The halophyte plant material was collected in September 2024 from its natural habitat at Ras El-Hekma shore, Mediterranean Coast, Egypt, 31.225543°N, 27.858790°E. Plants were identified by Amal M. Fakhry, Professor of Biodiversity, Department of Botany and Microbiology, Faculty of Science, Alexandria University. Voucher specimens were deposited in the Herbarium of Alexandria University (ALEX) at the Faculty of Science, deposition numbers ALEX 4123. The samples were air-dried and ground to a coarse powder. The powder was then added to distilled water in a weight-to-volume ratio of 1:20 (W/V) and placed in a water bath at 85 °C for 20 minutes. The fresh extracts were then filtered through double-layered cheesecloth and allowed to cool to room temperature (Salma et al., 2014). The resulting supernatant was taken as 100% *H. strobilaceum* water extract and diluted to 50% for usage as foliar spraying (Sofowora, 1982).

### 2.2 Plant materials, growth conditions, and treatments

Seeds of quinoa (Miser 1) were obtained from the Faculty of Agriculture, Alexandria University, Egypt. Homogenous seeds of the quinoa plant were sown in pots in a greenhouse of the Botany and Microbiology Department, Faculty of Science, Alexandria University, Alexandria, Egypt, during the winter season of 2024. The greenhouse conditions included average daytime temperatures of 20–25 °C, nighttime temperatures of 15–18 °C, relative humidity between 55–65%, and a natural photoperiod of approximately 10–11 hours of daylight. To prevent contamination, seeds were sterilized for two minutes in a 1% sodium hypochlorite solution while being shaken constantly. Ten seeds of quinoa were cultivated in plastic pots (30 cm diameter) filled with 7 kg of sandy soil. The experiment was conducted using a two-factor factorial design arranged in a completely randomized design (CRD) with five replicates, where the two factors were salinity levels (0, 25, 50, 75, 100, and 150 mM NaCl) and foliar spray treatments (distilled water and *H. strobilaceum* extract), resulting in a total of 60 pots. Fourteen days after germination, seedlings were thinned to three per pot, then the pots were irrigated with a constant concentration of salt (0, 25, 50, 75, 100, and 150 mM NaCl). The developed plants of both control and NaCl-treated pots were sprayed with *H. strobilaceum* extract after 14, 21, and 28 days of treatment with NaCl, while the untreated control plants were sprayed with distilled water only. *H. strobilaceum* extract and NaCl concentrations were selected based on our preliminary experiments. The experimental period extended from 17 November 2024 to 16 February 2025, corresponding to a total duration of 91 days (approximately 13 weeks). Salinity treatments was initiated 14 days after germination and maintained until the end of the experiment through regular irrigation with fixed NaCl concentrations; plant samples were collected after 8 weeks of sowing, while seeds were collected at the end of the experiment after 13 weeks. Plant samples were collected to analyze the following criteria.

### 2.3 Determination of plant growth parameters

The shoot height (cm), number of leaves, leaf area (cm^2^), root length, and dry weight of shoot and root, and seed yield were used as growth parameters. Randomly selected plants from each treatment were used to estimate plant growth parameters at the end of the experiment. The shoot height and root length of quinoa plants were manually measured using a measuring scale. Dry weights for shoot and root were measured after drying samples of fresh weights in the oven at 60 °C until constant weight. The image-processing program ImageJ ver. 1.53t was used to calculate the leaf area in cm^2^ (Martin et al., 2020; Schroeder et al., 2021). Additionally, seed yield weight was also assessed after complete growth and plant drying.

### 2.4 Determination of photosynthetic pigment content

Chlorophyll fluorescence was measured in fresh leaves using an OS-30P pulse-modulated chlorophyll fluorimeter (Opti-Sciences, Hudson, USA), following the method outlined by Van Kooten and Snel (1990). The maximum quantum efficiency ratio of PSII (Fv/Fm) was determined using an OS-30P pulse-modulated chlorophyll fluorimeter (Opti-Sciences, Hudson, USA).

### 2.5 Determination of osmolyte content

Soluble sugars from dry, powdered quinoa leaves were extracted using a borate buffer (pH 8.5). Following the method of DuBois et al. (1956). Sugar concentration was determined calorimetrically by mixing 0.1 ml of the borate extract with 3 ml of concentrated H_2_SO_4_ and 1 ml of 5% phenol. The mixture was incubated at 60 °C for 20 minutes, then cooled, and the absorbance was measured at 490 nm. A glucose standard curve was used to calculate the total soluble sugar content (mg/g dry matter).

Total protein content was determined following the method described by Pomory (2008). The working reagent was prepared by mixing 1% CuSO_4_·5H_2_O, 2% sodium potassium tartrate, and 2% Na_2_CO_3_ in a 1:1:100 ratio. A 0.1 mL aliquot of the extract was added to 3 mL of the working solution, briefly vortexed, and left to stand for 10 minutes. Then, 0.1 mL of 1 N Folin-Ciocalteu reagent was added, and the mixture was vortexed again. After 30 minutes, the absorbance was measured at 750 nm. Protein content was expressed as milligrams of bovine serum albumin (BSA) equivalents per gram of dry material (mg BSA/g DM).

Amino acid content was determined using the ninhydrin assay method described by Lee and Takahashi (1966), with glycine as the standard. For extraction, 0.1 g of finely powdered leaf tissue was mixed with 10 mL of 95% ethanol, and the resulting supernatant was used for analysis. A 0.1 mL aliquot of the plant extract or glycine standard was combined with 1.9 mL of a ninhydrin–citrate buffer–glycerol reagent, consisting of 0.5 mL of 1% ninhydrin in 0.5 M citrate buffer (pH 5.5), 1.2 mL of 55% (v/v) glycerol solution, and 0.2 mL of 0.5 M citrate buffer. The mixture was shaken and heated in a water bath at a boiling temperature for 12 minutes, then cooled. After mixing thoroughly, the absorbance was measured at 570 nm. Amino acid content was expressed as mg/g dry matter (DM), based on a glycine calibration curve.

Proline content was determined following the method of Bates et al. (1973). A 0.1 g sample of finely dried tissue was homogenized in 5 mL of 3% (w/v) aqueous sulfosalicylic acid, and the homogenate was centrifuged to remove residues. One milliliter of the supernatant was reacted with 2 mL of acid ninhydrin reagent—prepared by dissolving 1.25 g of ninhydrin in 30 mL of glacial acetic acid and 20 mL of 6 M phosphoric acid—and 2 mL of acetic acid. The mixture was incubated at 100 °C for 1 hour, and the reaction was then terminated by placing the tubes in an ice bath. The resulting chromophore was extracted with 4 mL of toluene, brought to room temperature, and its absorbance was measured at 520 nm. Proline concentration was calculated as mg/g dry matter (DM) using a standard curve prepared with proline.

### 2.6 Determination of ascorbic acid and total phenol content

Ascorbic acid, a non-enzymatic antioxidant, was quantified following the method of Oser and Hawk (1965). A 0.1 g sample of leaf tissue was homogenized in 5 mL of 5% (w/v) sulfosalicylic acid and centrifuged at 10,000 rpm for 10 minutes. The reaction mixture for ascorbic acid determination included 2 mL of 2% sodium molybdate, 2 mL of 0.15 N H_2_SO_4_, 1 mL of 1.5 mM Na_2_HPO_4_, and 1 mL of the tissue extract. The mixture was incubated in a water bath at 60 °C for 40 minutes, then cooled and centrifuged at 3,000 rpm for 10 minutes. Absorbance was recorded at 660 nm. Ascorbic acid content was expressed as mg/g dry matter (DM), calculated using a standard curve prepared with ascorbic acid.

Total phenolic content was quantitatively estimated using the method of Jindal and Singh (1975). A 0.1 g sample of dried tissue was extracted three times with 85% ethanol, and the resulting clear supernatants were pooled and adjusted to a final volume of 10 mL. One milliliter of this extract was mixed with 0.1 mL of Folin’s reagent and 1 mL of 20% Na_2_CO_3_, then diluted to 5 mL with distilled water. After 30 minutes, the absorbance was measured at 650 nm. Total phenolic content was calculated as mg/g dry matter (DM) using a gallic acid standard curve prepared using the same procedure.

### 2.7 Determination of hydrogen peroxide and malondialdehyde content

Hydrogen peroxide (H_2_O_2_) content in quinoa plants was measured following the method of Velikova et al. (2000). A 0.1 g sample of leaf tissue was homogenized in 5 mL of 0.1% trichloroacetic acid (TCA) and centrifuged at 12,000 rpm for 15 minutes. Subsequently, 0.5 mL of the supernatant was mixed with 0.5 mL of 10 mM potassium phosphate buffer (pH 7.0) and 1 mL of 1 M potassium iodide. The absorbance of the reaction mixture was recorded at 390 nm. H_2_O_2_ content was calculated using an extinction coefficient of 0.28 μM^-1^ cm^-1^ and expressed as μM per gram fresh matter (μM/g FM).

Lipid peroxidation in quinoa leaves was assessed by measuring the concentration of malondialdehyde (MDA), a byproduct of the peroxidation of unsaturated fatty acids (e.g., linolenic acid, 18:3), using the method of Heath and Packer (1968). A 0.5 g sample of fresh leaves was homogenized in 10 mL of 5% (w/v) trichloroacetic acid (TCA), and the mixture was centrifuged at 4,000 rpm for 15 minutes. Two milliliters of the resulting supernatant were combined with 2 mL of 0.67% (w/v) thiobarbituric acid (TBA) solution. The mixture was incubated in a boiling water bath at 100 °C for 20 minutes, then rapidly cooled. Absorbance was measured at 532 nm and corrected for nonspecific turbidity at 600 nm. MDA concentration was calculated using an extinction coefficient of 155 mM^-1^ cm^-1^ and expressed as micromoles per gram of fresh matter (μM/g FM).

### 2.8 Determination of mineral content

Wet digestion of dry, powdered leaf samples was performed using a mixture of 70% HNO_3_ and 30% H_2_O_2_ in a 4:2 (v/v) ratio. The concentrations of potassium (K), calcium (Ca), magnesium (Mg), and sodium (Na) in the digested samples were determined using an inductively coupled plasma optical emission spectrometer (ICP-OES) (Polyscan 61 E, Thermo Jarrell-Ash Corp., Franklin, MA, USA). Phosphorus (P) content was quantified colorimetrically using the molybdenum blue method with monopotassium phosphate as the standard. Nitrogen (N) was determined using the Rochelle reagent and ammonium chloride as a standard, following the procedure described by Allen et al. (1974).

### 2.9 Total lipid content

Lipids were extracted from 10 grams of ground quinoa seeds using n-hexane in a Soxhlet apparatus for 6 hours, following the procedure outlined by the AOCS (1998). The lipid content was calculated and expressed as milligrams per gram of dry matter (mg/g DM) for each sample.

### 2.10 SDS-PGE protein profile

Quinoa flour samples were defatted using hexane in a 1:3 (w/v) ratio for 24 hours at room temperature. Total proteins were extracted from seeds. Seed protein profiles were analyzed by SDS-PAGE (slab gel type) on a 10% polyacrylamide gel, following the method of Laemmli (1970). After electrophoresis, the gel was photographed, and protein bands were scored based on their presence (1) or absence (0). The molecular weights of the protein bands were estimated using Gel Analyzer software version 23.1.

### 2.11 Statistical analysis

The statistical analyses were conducted using IBM SPSS Statistics 27.0 (Crop, 2020). All results are expressed as mean ± standard deviation (SD) based on five replicates. The data were tested for compliance with ANOVA assumptions. The Shapiro–Wilk test was used to assess data normality, and Levene’s test was applied to verify the homogeneity of variances. The results confirmed that the data met the required assumptions for ANOVA analysis. A two-way analysis of variance (ANOVA) was then performed to assess the overall significance of treatment effects. *Post-hoc* tests were conducted to determine which specific treatment means differed; these are statistical procedures used after an initial analysis to determine which specific group differences are significant. They are conducted when the initial analysis indicates an overall significant effect but doesn’t specify which particular groups differ. Bonferroni-corrected t-tests were also applied. A Bonferroni-adjusted significance threshold of α = 0.0083 was used, corresponding to six pairwise comparisons.

## 3 Results

### 3.1 Impact of halophyte extract on morphological and yield traits of quinoa under salt stress

**Figure 1:**
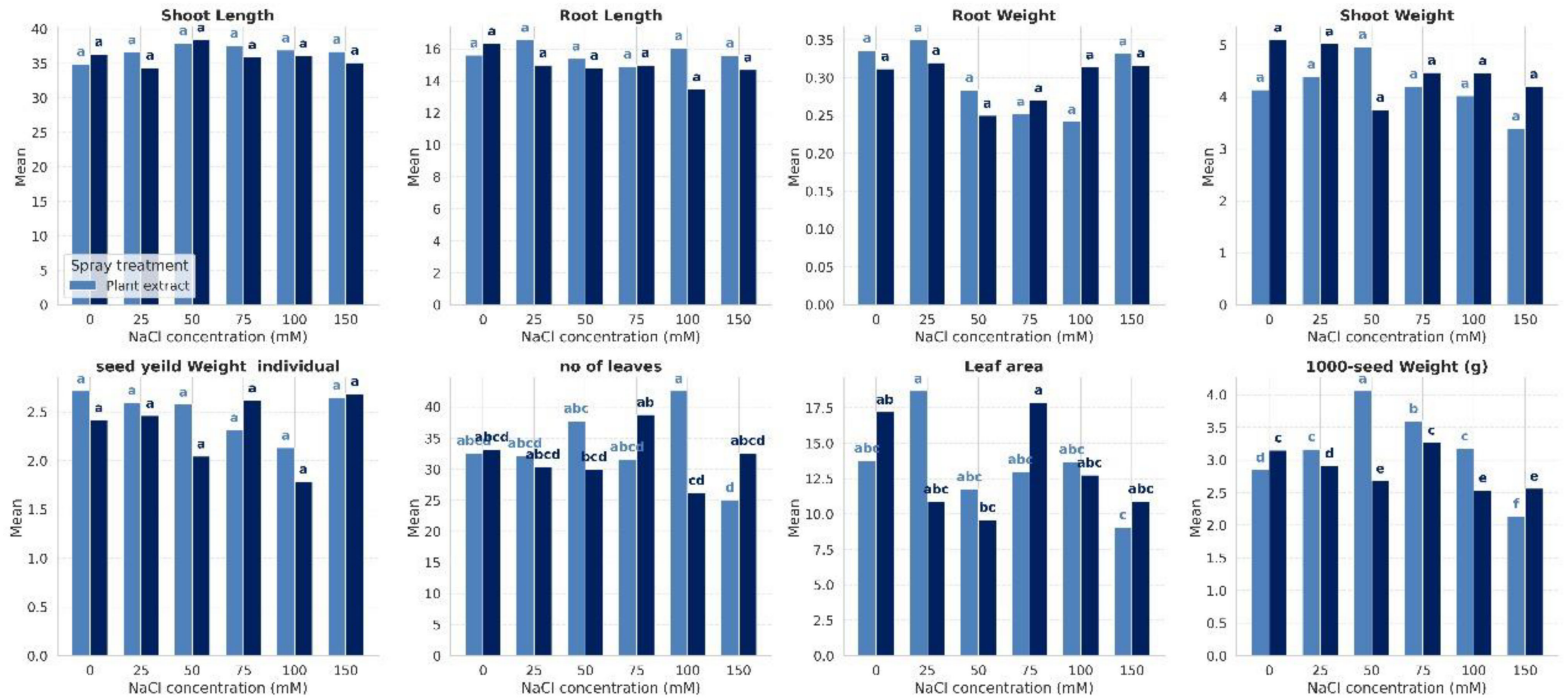
Effect of salinity and foliar spray treatment on the studied plant growth and yield traits across varying NaCl concentrations.

To assess the effectiveness of *Halocnemum strobilaceum* extract (HE) in improving quinoa growth performance under saline conditions, we evaluated several growth-related parameters, including shoot and root lengths, dry weights of shoots and roots, number of leaves, leaf area, and seed yield. These measurements were conducted under both non-stressed and salt-stressed conditions, with and without foliar application of *H. strobilaceum* extract (**Figure 1**). The data revealed that increasing salinity was generally associated with a reduction in most growth and yield traits, indicating that salinity stress had a substantial negative impact on vegetative growth components. Notably, Plant extract helped maintain higher shoot and root length at moderate to high NaCl (75-100–150 mM). It is also notable that salt stress led to a reduction in both shoot and root weights across all treatments. However, under the foliar spray treatments, a marked increase in shoot weight was observed at 50 mM NaCl, while root weight showed a considerable increase at 150 mM NaCl. Results also revealed that plant extract is especially effective at maintaining leaf number under high (100 mM) salt stress, while at 25 mM NaCl, it maintained a much larger leaf area as compared to the control. The plant extract was most effective at moderate salinity levels (particularly at 50 mM NaCl), where it helped sustain or even enhance seed weight compared to both low (0–25 mM) and high (150 mM) salinity conditions. However, for seed yield per individual plant, the extract was most beneficial under both 100 mM and 50 mM salinity levels.

The statistical analysis revealed significant differences among treatments in response to salinity stress and foliar application of the halophyte extract. Traits such as shoot and root length and shoot and root weight showed no significant variation between plant extract and distilled water treatments. However, yield attributes (1000-seed weight, leaf area, and number of leaves) were significantly (p≤ < 0.05) enhanced under extract treatment at higher salinity levels, while distilled water-treated plants showed considerable declines, reflected by separation into distinct significance groups.

We performed a two-way ANOVA with interaction effects on the row data of our treatments. The complete results of ANOVA for the eight variables of the quinoa plant were presented in **Table 1**. The results revealed that leaf area was significantly (<0.05) affected by NaCl concentration and by the interaction between NaCl and foliar spray treatment. The maximum reduction (-44.3%) in leaf area was noticed at 50 mM NaCl, followed by 25 mM NaCl (-36.8%) and 150 mM NaCl (-36.6%). Besides, the combination of NaCl concentration and spray treatment significantly affected leaf number, even though neither factor alone did. It is also notable that both NaCl concentration and spray treatment have a highly significant (<0.001) impact on the weight of seeds. Moreover, a significant interaction (<0.001) between the two factors indicates that the effect of the spray treatment varies depending on the level of salinity. Results revealed that salinity significantly reduces the seed weight, especially under 100 mM NaCl, resulting in a reduction of -19.4% in the weight of 1000 seeds and a decrease by -26.4% in the weight of individual seeds, as compared to the control. Foliar spray of HE at 50 mM NaCl resulted in a +33.9% increase in the weight of 1000 seeds and a +20.5% rise in the weight of individual seeds, as compared to stressed plants under the same salinity level (**Figure 1**).

**Table 1:** ANOVA probability (p) values for the effects of *Halocnemum strobilaceum* extract (HE) spray treatment, salinity level, and their interaction on the growth and yield traits of quinoa plants (Values ≤ 0.05 indicate significant differences).

Source of variation	Shoot length	Root length	Root weight	Shoot weight	Individual seeds yield weight	Leaves number	Leaf area	Weight of 1000 seeds
HE Spray treatment	0.503	0.121	0.907	0.363	0.355	0.237	0.897	<0.001
Salinity treatment (mM NaCl)	0.747	0.707	0.121	0.709	0.233	0.166	0.002	<0.001
Interaction	0.928	0.495	0.619	0.503	0.774	<0.001	0.005	<0.001

### 3.2 Impact of salt stress on physiological and biochemical parameters of quinoa plant

Data in **Table 2** showed the main strategies that the quinoa plant uses to handle salt stress. The metabolic peaks were as follows: sugars: 25 mM (49.556), proteins: 75 mM (56.698), proline: 100 mM (13.736), and amino acids: 100 mM (73.166). The oxidative stress markers revealed that H_2_O_2_: highest at 150 mM (109.480), and MDA: highest at 25 mM (95.015). For ion homeostasis, Na^+^ influx: 50 mM (0.130), K^+^ depletion: 50 mM (5.072), and Ca²^+^ accumulation: 100 mM (0.325). However, photosynthetic efficiency: Fv/Fm: is highest at 150 mM (0.7547), and Fv/Fo: is highest at 100 mM (3.1179). It is also noted that recovery occurred at high salinity (150 mM): improved lipids (4.485), Fv/Fm (0.7547), and Fv/Fo (3.0864), and partial restoration of sugars (39.104) and proteins (55.426).

**Table 2:** Means and standard deviations of the studied variables of the *Chenopodium quinoa* plant under different treatments.

Variable	Tap water	25 mM NaCl	50 mM NaCl	75 mM NaCl	100 mM NaCl	150 mM NaCl
Sugars	43.195 ± 8.537	49.556 ± 3.501	48.897 ± 5.029	27.577 ± 5.642	29.467 ± 5.297	39.104 ± 8.546
Proteins (mg g-1 DM)	53.540 ± 1.629	55.844 ± 1.068	55.469 ± 2.173	56.698 ± 8.675	52.516 ± 6.482	55.426 ± 0.806
Phenols (mg g-1 DM)	27.435 ± 1.383	30.025 ± 0.713	27.667 ± 0.890	25.709 ± 1.647	24.277 ± 0.793	24.024 ± 0.584
Proline (mg g-1 DM)	6.848 ± 0.862	8.905 ± 1.053	8.745 ± 0.608	10.010 ± 0.799	13.736 ± 0.286	9.483 ± 1.711
H_2_O_2_ (μM g-1 FM)	102.247 ± 23.028	87.220 ± 15.789	99.447 ± 2.193	77.467 ± 16.585	95.387 ± 14.001	109.480 ± 18.562
Amino Acids (mg g-1 DM)	25.480 ± 7.373	27.854 ± 4.402	36.262 ± 2.937	44.477 ± 20.670	73.166 ± 2.809	49.226 ± 3.123
Ascorbate (mg g-1 DM)	29.597 ± 0.744	29.506 ± 0.521	29.354 ± 0.359	30.029 ± 1.019	28.852 ± 0.745	29.567 ± 2.558
MDA (μM/g FM)	62.853 ± 5.402	95.015 ± 35.053	81.375 ± 6.984	61.923 ± 2.781	85.638 ± 25.095	86.800 ± 7.845
N (mg/g)	4.774 ± 0.088	5.075 ± 0.087	4.613 ± 0.256	4.748 ± 0.273	4.847 ± 0.167	5.380 ± 0.252
P (mg/g)	0.463 ± 0.019	0.470 ± 0.034	0.490 ± 0.014	0.475 ± 0.010	0.485 ± 0.019	0.475 ± 0.029
K (mg/g)	6.140 ± 0.582	5.225 ± 0.247	5.072 ± 0.411	5.148 ± 0.167	5.377 ± 0.082	4.925 ± 0.083
Ca (mg/g)	0.270 ± 0.009	0.315 ± 0.019	0.235 ± 0.010	0.265 ± 0.010	0.325 ± 0.010	0.305 ± 0.019
Mg (mg/g)	0.220 ± 0.045	0.220 ± 0.024	0.225 ± 0.019	0.225 ± 0.019	0.220 ± 0.024	0.250 ± 0.014
Na (mg/g)	0.085 ± 0.010	0.068 ± 0.025	0.130 ± 0.055	0.063 ± 0.019	0.093 ± 0.020	0.040 ± 0.026
Seed Lipids (g/100g)	5.012 ± 0.593	3.837 ± 0.362	3.868 ± 0.315	3.413 ± 0.214	3.557 ± 0.155	4.485 ± 0.094
Fo	186.17 ± 2.79	156.00 ± 2.97	167.33 ± 32.87	151.33 ± 31.77	147.17 ± 15.17	215.83 ± 37.43
Fv	501.00 ± 52.59	459.00 ± 32.87	401.33 ± 73.03	459.83 ± 189.33	466.67 ± 138.39	660.00 ± 73.03
Fm	687.17 ± 49.85	612.33 ± 33.60	568.33 ± 105.89	610.67 ± 221.28	613.67 ± 154.09	876.00 ± 110.65
Fv/Fm	0.7276 ± 0.0238	0.7490 ± 0.0126	0.7067 ± 0.0034	0.7404 ± 0.0418	0.7528 ± 0.0365	0.7547 ± 0.0120
Fv/Fo	2.6951 ± 0.3223	2.9399 ± 0.1561	2.4043 ± 0.0365	2.9272 ± 0.6367	3.1179 ± 0.6196	3.0864 ± 0.1969

The values are means ± SD.

More specifically, results showed that quinoa responses to salinity are concentration-dependent and non-linear (**Table 2**). This suggests salt concentration critically influences sugar dynamics, with 25 mM being a potential optimum for maximizing sugar levels, which drop sharply at 75–100 mM, and partially recover at 150 mM. The protein peak was at 75 mM, suppressed at 100 mM. and a partial restoration is noticed at 150 mM. On the other hand, phenols showed a salt concentration-dependent decline, as they decreased steadily from 25 mM to 150 mM. Massive proline accumulation at 100 mM NaCl confirms its function as a key osmolyte under productivity-limiting saline conditions, but our data showed a sharp decline from 100 mM (13.74) to 150 mM (9.48). Effective reactive oxygen species (ROS) detoxification was noticed at 25 mM and 75 mM, showing the lowest H_2_O_2_ (87.22 and 77.47, respectively). This coincides with metabolic shifts: at 25 mM, peak phenols and sugars, and at 75 mM, peak proteins. while salinity of 150 mM represents a critical threshold for cellular damage, showing the highest H_2_O_2_ value (109.48) in our experiment. Notable progressive accumulation of amino acids with increasing salinity levels was detected, while at 150 mM, the concentration of amino acids dropped from 73.17 to 49.23. The data reveal 75 mM NaCl as the optimal concentration for ascorbate-mediated antioxidant defense (30.029), while 100 mM represents a critical threshold for antioxidant system failure, where ascorbate decreased to its minimal value (28.852). Malondialdehyde (MDA) is considered a marker of oxidative damage to lipids. Data of lipid peroxidation under salt stress in **Table 2** showed that minimal damage was at 75 mM NaCl (61.92), with -1.5% vs control. The 75 mM NaCl concentration uniquely combines the lowest oxidative damage (MDA) with peak metabolic performance (proteins, ascorbate), making it the optimal stress-adaptation zone. A comprehensive analysis of mineral nutrient responses to salt stress in our experiment showed a classic salt stress response at 50 mM NaCl, where Na+ influx (0.13 vs 0.085 control) coincides with K+ depletion (5.07 vs 6.14 control), parallel to Ca²^+^ suppression (0.235). On the other hand, the K^+^/Na^+^ Ratio (Salinity Tolerance Indicator) collapses at 50 mM (39.02 vs 72.24 control) with a drop of -46%. Recovery at 150 mM (123.13) via aggressive Na^+^ exclusion despite K^+^ depletion. Ca²^+^/Na^+^ Ratio (Membrane Protection Marker) had its peak at 100xmM (3.49), coinciding with maximal Ca²^+^ accumulation (0.325) and salt overly sensitive (SOS) pathway activation, which is a major signaling pathway in plants that helps them tolerate salt stress. All salt treatments reduce lipids by 10-32% vs control (**Table 2**). The maximal lipid reduction was noticed at 75 mM NaCl (-31.9%), coinciding with the lowest sugars (27.58), and oxidative stress (MDA: 61.92). Partial recovery was notable at high salinity:150 mM NaCl, which showed the highest lipid content (4.485) among salt-treated groups. This, in turn, matches a recovery in sugars (39.10) and proteins (55.43). Lower variability (SD = 0.094) suggests a consistent response. Chlorophyll fluorescence parameters under salt stress revealed that 50 mM NaCl is the most disruptive, showing the lowest Fv/Fm (0.7067) and Fv/Fo (2.4043), while 150 mM NaCl showed unexpected resilience, attaining the highest Fv/Fm (0.7547). PSII vitality (Fv/Fo) attained the peak performance at 100 mM (3.1179) and 150 mM (3.0864). This analysis identifies 50 mM NaCl as the critical photoinhibition threshold and 100–150 mM as the zone of metabolic compensation where improved PSII vitality (VR) offsets ROS accumulation.

### 3.3 Impact of halophyte extract on physiological and biochemical parameters of quinoa under salt stress

The foliar application of *H. strobilaceum* extract (HE) demonstrated significant modulatory effects on biochemical and physiological parameters compared to the distilled water spray (DW). Statistical validation of *H. strobilaceum* extract’s salinity mitigation effects was carried out using two-way ANOVA with interaction effects, followed by Bonferroni-corrected t-tests on the row data of our treatments. The complete results of ANOVA for all 20 variables were presented in **Table 3**. The results revealed that 15 out of 20 variables show salinity-dependent responses to *H. strobilaceum* extract.

**Table 3:** Interaction effect of *Halocnemum strobilaceum* extract (HE) spray and salinity on measured variables (Two-Way ANOVA).

Variable	Interaction p-value	Interpretation
Sugars	< 0.001	Strong interaction
Proteins	0.002	Significant
Phenols	0.532	Not significant
Proline	< 0.001	Strong interaction
H_2_O_2_	0.003	Significant
Amino acids	0.001	Significant
Ascorbate	0.890	Not significant
MDA	< 0.001	Strong interaction
N	0.021	Significant
P	0.045	Significant
K	0.624	Not significant
Ca	0.035	Significant
Mg	0.210	Not significant
Na	0.001	Significant
Seed lipids	0.038	Significant
Fo	0.570	Not significant
Fv	0.008	Significant
Fm	0.012	Significant
Fv/m	0.008	Significant
Fv/o	< 0.001	Strong interaction

Significant interactions (p ≤ 0.05) confirm HE’s effects depend on salinity concentration.

The ANOVA results indicated the overall significant difference among our treatments. To determine which treatment means differed, *post-hoc* comparisons were performed. The mitigation effects of *H. strobilaceum* extract under critical salinity levels were further evaluated using Bonferroni-corrected t-tests. Variables showing significant interactions are summarized in **Table 4**, applying a Bonferroni-adjusted significance threshold of α = 0.0083 for six comparisons. Mitigation effect size was calculated as: Mitigation (%) = (1 - (Mean_HE/Mean_DW)) * 100 and Improvement (%) = ((Mean_HE - Mean_DW)/Mean_DW) * 100.

**Table 4:** *Halocnemum strobilaceum* extract’s effects at critical salinity levels (post-Hoc t-Tests) HE, *H. strobilaceum* extract spray; DW, Distilled water spray. (p-values ≤ 0.05 indicate significant differences).

Variable	NaCl (mM)	DW mean ± SD	HE mean ± SD	Mitigation/Improvement	p-value	Sig. (α=0.0083)
Sugars	25	46.73 ± 0.31	52.38 ± 2.49	-12.1%	0.012	No
	50	53.28 ± 2.05	44.51 ± 0.69	16.5%	0.001	Yes
	100	33.94 ± 2.65	24.99 ± 1.67	26.4%	0.006	Yes
Proteins	75	64.35 ± 2.41	49.04 ± 1.79	23.8%	< 0.001	Yes
	100	46.60 ± 0.17	58.43 ± 0.21	25.4%	< 0.001	Yes
Proline	25	9.61 ± 1.09	8.20 ± 0.08	14.7%	0.032	No
	150	11.02 ± 0.43	7.95 ± 0.05	27.9%	< 0.001	Yes
H_2_O_2_	75	92.59 ± 0.98	62.35 ± 0.84	32.7%	< 0.001	Yes
	150	126.37 ± 1.13	92.59 ± 1.96	26.7%	< 0.001	Yes
MDA	25	126.95 ± 2.33	63.09 ± 2.69	50.3%	< 0.001	Yes
	100	106.49 ± 15.78	64.79 ± 4.42	39.2%	0.007	Yes
N	150	5.61 ± 0.01	5.15 ± 0.02	8.2%	< 0.001	Yes
Seed lipids	0	4.46 ± 0.01	5.54 ± 0.01	24.2%	< 0.001	Yes
	50	3.57 ± 0.01	4.16 ± 0.01	16.5%	< 0.001	Yes
Fv/o	100	2.55 ± 0.01	3.68 ± 0.03	44.3%	< 0.001	Yes
	150	2.91 ± 0.01	3.27 ± 0.01	12.4%	< 0.001	Yes
	150	2.91 ± 0.01	3.27 ± 0.01	12.4%	< 0.001	Yes

The results presented in **Table 4** revealed that foliar application of *H. strobilaceum* extract (HE) significantly enhanced the photosynthetic efficiency (Fv/o) of quinoa plants, with improvements of 44.3% at 100 mM and 12.4% at 150 mM NaCl. This enhancement was accompanied by a marked reduction in oxidative stress, as evidenced by significant decreases in hydrogen peroxide (H_2_O_2_) levels at 75 mM (32.7%) and 150 mM (26.7%) (p < 0.001). Furthermore, lipid peroxidation, indicated by malondialdehyde (MDA) content, was significantly mitigated at 25 mM (50.3%) and 100 mM (39.2%), confirming the extract’s protective effect against oxidative damage. This coincided with a significant increase in protein (25.4%) at 100mM NaCl (p < 0.001). Notably, seed lipid content showed an increase of 24.2–16.5% under 0 mM NaCl and 50 mM treatments, respectively. Conversely, phenols, ascorbate, K, Mg, and Fo showed no consistent response to foliar application of *H. strobilaceum* extract (**Table 4**).

### 3.4 Effects of halophyte extract on quinoa seed protein expression under salt stress

**Figure 2:**
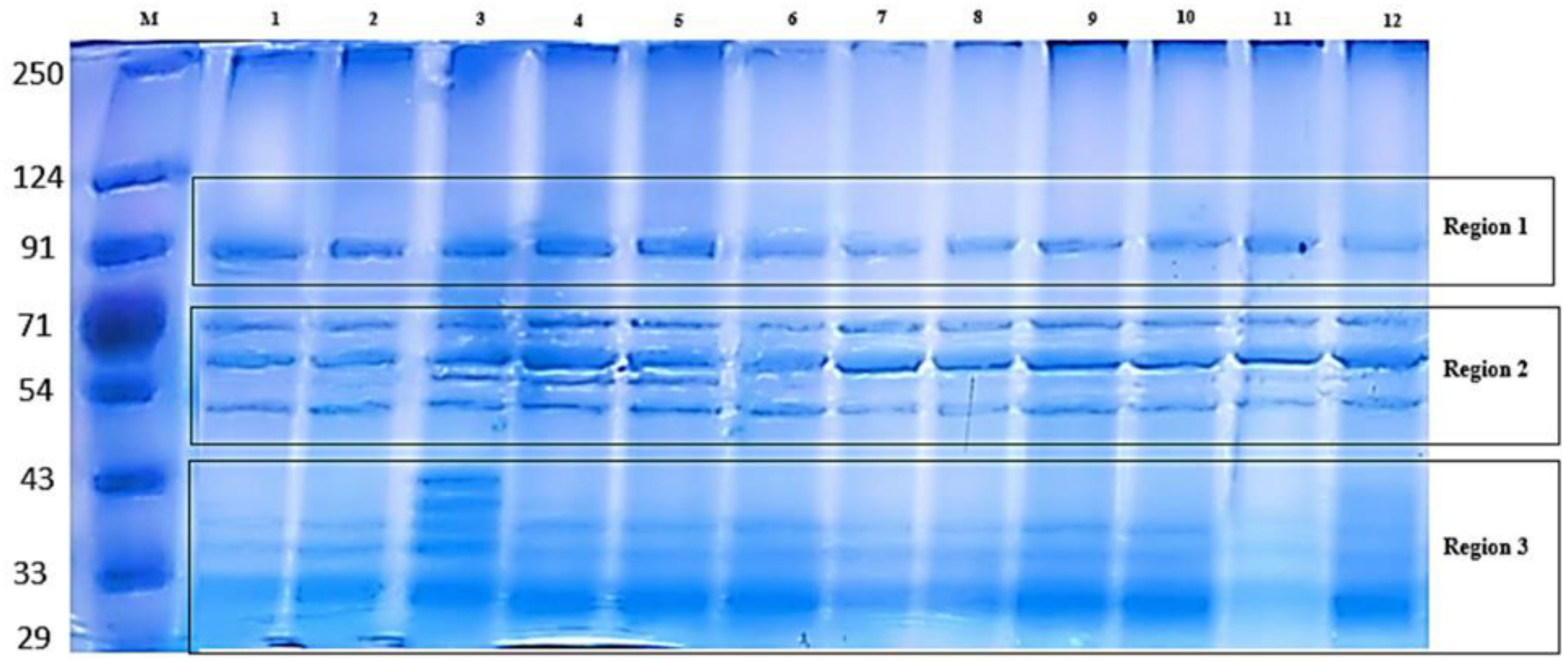
Sodium Dodecyl Sulfate–Polyacrylamide Gel Electrophoresis (SDS-PAGE) protein electrophoretic patterns of treated *Chenopodium quinoa*. The Lanes showed M: Marker (LMW from 29–250 KDa) (See **Figure 3** for detailed code numbers of different treatments).

**Figure 3:**
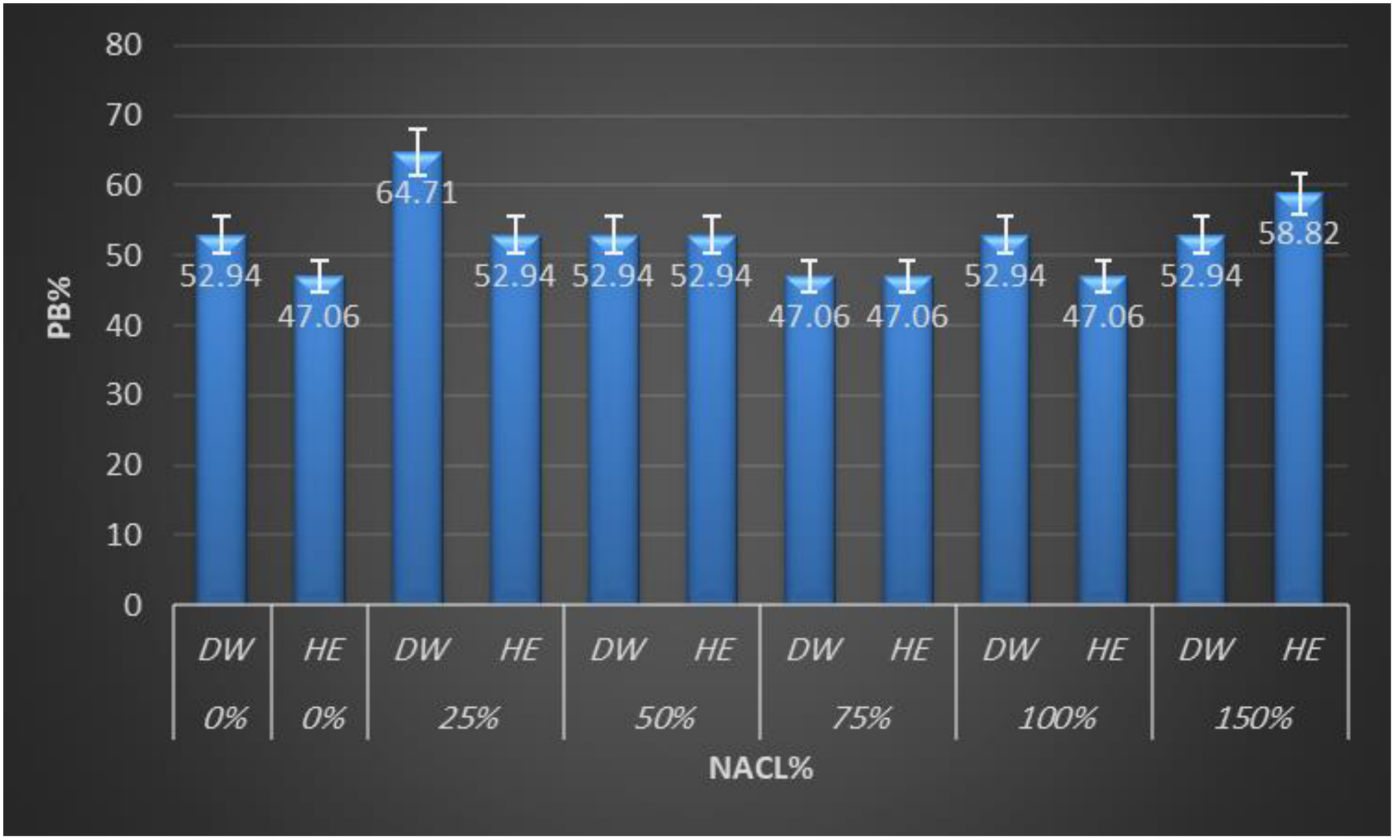
Clustered bar chart from Sodium Dodecyl Sulfate–Polyacrylamide Gel Electrophoresis (SDS-PAGE) analysis of treated *Chenopodium quinoa* plants. DW, distilled water; HE, *Halocnemum strobilaceum* extract.

Total protein was extracted from quinoa seeds and separated by SDS-PAGE to visualize seed protein banding patterns among treatments. This electrophoretic technique enables the separation of complex protein mixtures based on molecular weight and allows the detection of proteins at microgram levels. The resulting profiles indicated observable variations in band intensity and presence/absence among treatments under saline conditions. However, the SDS-PAGE analysis presented here provides only a qualitative overview of protein pattern differences and does not allow precise identification or quantification of the individual proteins. The electrograms exhibited distinctive quantitative and qualitative alterations. The electrophoretic pattern of total quinoa proteins from five different salinity treatments on SDS-polyacrylamide gel is shown in **Figure 2**. Variations can be observed in the quinoa protein; three regions, designated as 1, 2, and 3, were observed with distinctions among and between them. Variations were shown in the 2nd and 3rd regions. As shown in **Figure 2**, total soluble proteins in all treatments were resolved into 17 bands ranging from 8 to 12 bands. All treatments showed polymorphic bands at molecular weights ranging from 250 KDa to 29 KDa. Seed storage quinoa protein revealed eight monomorphic/common bands at molecular weights (250, 91, 71, 60, 48, 39, 34, and 31KDa) in all treatments. Herein, there are four main known quinoa protein/subunits, namely albumins (can’t be detected), globulins (34, 39 KDa), glutelins (60, 71, 91 KDa), and prolamins (31 KDa). Quinoa protein is rich in globulins and albumins more than prolamins. Stress-induced variations in protein expression are reflected in the presence or absence of particular bands at increasing NaCl concentrations. One band (54 KDa in 150 treatments) and two bands (40, 43 KDa in 25%) disappeared. Plant extract was unable to mitigate the effects of NaCl stress at both mild and high salinity percent (25% and 100%), where decreased the number of bands to 9 and 8 bands compared to their water control (11 &9 bands), reflected on low polymorphism (Pb= 52.94, 47.06%) than its water control (pb%= 64.71, 52.94%) as shown in **Figure 3**. Notably, both salinity treatments of 50% and 75% showed a constant band number and percent of polymorphism, like their water control. The 50% treatment revealed 9 bands with high Pb% %=52.94%, while the 75% treatment showed 8 bands with low polymorphism (Pb=47.06%). In contrast, 150% of NaCl seems to mitigate the effects of NaCl stress somewhat, as seen by the two novel protein bands that were found at 43 and 30 KDa in comparison to tap water. The greatest polymorphism was evident in the growing number of bands (10 Pb% bands = 58.82 vs. 9 control bands of Pb% 52.94%). The study indicated that some unique bands appear only with extract treatment at 150 mM NaCl reflecting potential qualitative alterations in the protein profile that warrant future molecular validation.

## 4 Discussion

Quinoa (*Chenopodium quinoa*) is widely acknowledged as a strategic crop for saline and arid agroecosystems owing to its inherent tolerance to abiotic stresses, high nutritional value, and adaptability to marginal environments (Taaime et al., 2023; Zouari et al., 2025). Improving quinoa performance under salinity is therefore a key target for enhancing food security in regions facing soil and water salinization. It is important to emphasize that salinity tolerance in *Chenopodium quinoa* is strongly genotype-dependent with marked variations among cultivars and ecotypes in ion regulations, osmotic adjustment, photosynthetic stability, and yield responses (Ruiz-Carrasco et al., 2011; Bazile et al., 2016; Abdelazeem et al., 2025). Comparative studies of quinoa accessions from different Chilean regions have also demonstrated pronounced physiological and molecular variability under NaCl stress (Ruiz-Carrasco et al., 2011). The genetic variability of quinoa is huge, with cultivars of quinoa being adapted to growth from sea level to 4000 masl, from 40 degrees south to 2 degrees north latitude, and from cold, highland climate to subtropical conditions. This makes it possible to select, adapt, and breed cultivars from a wide range of environmental conditions (Jacobsen, 2003). The results of Cai and Gao (2020) further indicate that salinity tolerance in quinoa is mainly associated with efficient leaf osmoregulation, potassium ion retention and sodium ion exclusion, and overall ion homeostasis rather than enhanced antioxidative capacity. This framework aligns with foundational halophyte ecophysiology studies (Koyro et al., 2008) and established models of ionic homeostasis in quinoa, which highlight the critical role of Na^+^/H^+^ antiporters and osmotic adjustment (Shabala et al., 2013; Adolf et al., 2013). Accordingly, the K^+^/Na^+^ ratio in leaves or roots may serve as a reliable physiological marker for selecting and breeding salt-tolerant quinoa cultivars. Therefore physiological responses of *C. quinoa* can not be generalized at the species level, and our results obtained for the Miser-1 cultivar should be interpreted within this framework of genetic variability. Although quinoa is generally considered moderately salt-tolerante, its adaptive responses vary considerably within genotype and environmental conditions. Recent strategies to sustain quinoa yields have focused on genotype selection, optimized agronomic practices, and the use of biostimulants and microbial inoculants. Biostimulants comprise organic or inorganic substances and/or beneficial microorganisms that, when applied to plants, activate a range of physiological and biochemical processes, thereby enhancing growth, productivity, and resilience to environmental stresses (Ali et al., 2021; Franzoni et al., 2022; Ma et al., 2022). Consistently, Hajihashemi et al. (2022) previously documented that quinoa’s tolerance to salinity can be strengthened through biochemical priming approaches. Specifically, treatments with salicylic acid and sodium nitroprusside enhanced antioxidant defense mechanisms, mitigated oxidative damage, and promoted osmoprotectant accumulation, thereby improving its resilience to salinity stress. Slatni et al. (2024) showed that the use of plant growth-promoting rhizobacteria isolated from halophytic rhizospheres, particularly *Bacillus* species, were found to enhance quinoa growth and salinity tolerance by improving root traits, biomass, and physiological performance. In line with these innovative approaches, our results indicate that foliar application of *H. strobilaceum* extract (HE) induced physiological and biochemical adjustments in quinoa, including modulation of osmolyte levels and chlorophyll fluorescence parameters, suggesting its potential as a natural biostimulant to improve crop resilience under saline conditions. It is noteworthy that in the present study, the responses of quinoa at 25 and 75 mM NaCl were generally similar to those observed at 50 and 100 mM, respectively. Nevertheless, these intermediate concentrations were included to establish a finer gradient of salinity stress. This approach allowed for a more consistent assessment of quinoa’s tolerance, although future investigations may benefit from focusing on fewer but more informative salinity levels to enhance clarity.

Elevated salinity levels were known to significantly suppress quinoa growth traits, including root length, leaf area, and seed weight, confirming the adverse impact of salt stress on both vegetative growth and yield components (Liu et al., 2024). In our study, salinity imposed a strong inhibitory effect on quinoa growth, as shown by reductions in root length (−17.4%), leaf area (−44.3%), and seed weight (−19.4% for 1000-seed weight and −26.4% for individual seed weight). The foliar application of HE mitigated these negative effects, particularly at 50–100 mM NaCl, where the number of leaves, leaf area, and seed weight were substantially improved compared to untreated plants. Notably, **HE application** enhanced seed weight by up to 33.9% and leaf area by 8.6%, thereby supporting yield stability even under maximum tested salinity levels. These findings are consistent with field studies in Egypt (Ali et al., 2023), where the combined application of potassium fertilization and foliar spraying of seaweed extract markedly improved quinoa yield and grain quality across two growing seasons, underscoring the potential of biostimulant-based strategies to enhance crop productivity under challenging environments.

Salinity stress hinders quinoa performance, causing declines in photosynthetic rate, leaf area, and biomass in a dose‐dependent manner (Slatni et al., 2024; Slimani et al., 2022). In the present study, quinoa’s physiological response to salinity followed a concentration‐dependent pattern: at low salinity (25 mM), osmotic adjustment occurred via increased sugars, proteins, and phenols — but lipid peroxidation also indicated early oxidative stress; at moderate levels (50–75 mM), metabolic dysregulation became evident, including photoinhibition (Fv/Fm ≈ 0.71) and K^+^/Na^+^ imbalance; while at high levels (100–150 mM), plants shifted toward hyperosmotic adaptation with elevated proline, amino acids, and Ca²^+^ accumulation to preserve membrane integrity. Notably, Fv/Fm values remained within the optimal range across salinity treatments, with a slightly higher value at 150 mM NaCl (0.7547) compared with the control (0.7276), indicating no severe photoinhibition of PSII. However, stable Fv/Fm does not imply the absence of salinity stress. In the quinoa cultivar Miser-1, PSII efficiency was maintained while growth and yield declined, suggesting that yield reduction under high salinity is mainly associated with morphological constraints, particularly reduced leaf area, rather than photochemical damage. This indicates a protective adjustment that preserves PSII function under stress at the expense of productivity. A similar pattern has been reported in quinoa under other abiotic stresses. For example, Sven-Erik Jacobsen et al. (2005) showed that frost stress can markedly reduce quinoa productivity even when the photosynthetic apparatus remains relatively functional, indicating that yield losses may arise from stress-induced limitations on growth and development rather than direct damage to photosynthesis. Likewise, in the present study, salinity reduced productivity despite the maintenance of PSII efficiency, suggesting that yield decline is more closely associated with morphological and growth constraints than with photochemical impairment. Foliar application of HE at 0 mM NaCl (control) significantly increased seed lipid content (p<0.001), indicating that the extract contains bioactive compounds that can stimulate baseline metabolic processes even in the absence of salt stress. At a salinity level of 50 mM NaCl, the extract maintained seed lipid accumulation and moderated sugar decline, suggesting preservation of primary carbon metabolism and a capacity to buffer mild osmotic/ionic perturbation; similar protective effects of foliar biostimulants and plant extracts on carbohydrate and seed quality under mild salt stress have been reported in several crop species (Ma et al., 2022). At 100 mM NaCl, *H. strobilaceum* extract produced the most pronounced functional benefits: significantly increased protein content and photochemical efficiency (Fv/o) while significantly lowering MDA (p<0.001), Collectively indicating enhanced photosynthetic stability and reduced lipid peroxidation under the applied saline conditions. At the highest level tested (150 mM NaCl), the extract continued to attenuate stress biomarkers by reducing H_2_O_2_ and proline accumulation and by modestly improving photochemical performance, which implies a capacity to limit excessive ROS accumulation and to prevent over-accumulation of osmolytes that can indicate metabolic distress. These improvements align with previous studies of Hajihashemi et al. (2022), who proved that salicylic acid and sodium nitroprusside, as a nitric oxide donor, priming significantly improved the antioxidant defense systems in boosting salinity tolerance in quinoa plants, and significantly reduced superoxide dismutase activity, which was accompanied by a significant decrease in hydrogen peroxide accumulation under salinity stress (100 mM NaCl). Flowers and Colmer (2008) reported that the unexpected resilience at 150 mM NaCl highlights species-specific adaptations, where Na^+^ exclusion and nitrogen mobilization counterbalance energy deficits—a strategy commonly observed in halophytes but rarely in glycophytes. In our study, HE notably increased protein content and seed lipid accumulation, even under saline conditions, indicating that it not only alleviates stress but also helps maintain grain nutritional quality.

The SDS-PAGE analysis provided a preliminary qualitative overview of quinoa seed protein patterns and suggested that saline conditions were associated with observable variations in band intensity and presence/absence among treatments. Across treatments, eight monomorphic bands represented major storage proteins, including globulins (34, 39 kDa), glutelins (60, 71, 91 kDa), and prolamins (31 kDa). Loss of specific bands (e.g., 54 kDa at 150 mM NaCl; 40 and 43 kDa at 25 mM NaCl) suggests stress-induced protein denaturation or repression of synthesis (Zhang et al., 2013). Conversely, the emergence of two novel bands under high salinity (150 mM NaCl + HE) indicates possible *de novo* synthesis of stress-related proteins, a phenomenon reported in halophytes under high salinity (Flowers and Colmer, 2008). The reappearance of those two novel protein bands at 43 and 30 kDa in the protein profile of HE-treated salinized quinoa plants, which were missing in the protein profiles of untreated salinized plants, may result from the partial suppression of the gene(s) responsible for these particular proteins (Ibrahim et al., 2014), or could be due to improved growth and elevated osmolyte levels (Abdel Latef et al., 2017; Hussein et al., 2019; Ibrahim et al., 2014). These findings support the role of HE in modulating quinoa’s proteomic response, particularly at high salinity levels.

Overall, our findings demonstrate that *H. strobilaceum* extract (HE) is associated with improved physiological performance and maintenance of grain nutritional quality in *C. quinoa* grown under saline conditions. The extract enhances quinoa’s salinity tolerance through modulation of physiological, biochemical, and proteomic responses, highlighting its potential as a natural biostimulant. Although the greenhouse-based conditions may not fully extrapolate to field environments, these results provide a robust foundation for future field validation and mechanistic investigations, supporting HE as a promising strategy for quinoa cultivation in saline environments.

## 5 Conclusions

Foliar application of *Halocnemum strobilaceum* extract (HE) was associated with improvements in several physiological and yield-related traits of quinoa under saline conditions. Specifically, HE increased seed weight by up to 33.9%, improved leaf area by 8.6%, enhanced protein content and photochemical efficiency (Fv/Fm), and reduced oxidative stress markers (MDA and H_2_O_2_). Its protective role was particularly evident at high salinity levels, where HE promoted seed nutritional quality, preserved membrane integrity, and induced the synthesis of stress-related proteins, demonstrating its association with improved yield-related traits and the maintenance of grain nutritional quality. Collectively, these findings indicate that HE functions as a sustainable natural biostimulant capable of supporting quinoa productivity under productivity-limiting saline conditions. For practical application, further research is needed to validate the HE efficacy under field conditions, and explore its effects across a broader range of salinity levels (300–500 mM NaCl).

## Data Availability

The original contributions presented in the study are included in the article/supplementary material. Further inquiries can be directed to the corresponding authors.
