# Correction: Foliar application of *Halocnemum strobilaceum* improves *Chenopodium quinoa* growth and physiological traits for saline agricultural residence

**DOI:** 10.3389/fpls.2026.1830445

**Published:** 2026-05-25

**Authors:** Ghalia S. Aljeddani, Amal M. Fakhry, Ameina S. Almoshadak, Soliman M. Toto

**Affiliations:** 1 Department of Environmental Sciences, College of Science, University of Jeddah, Jeddah, Saudi Arabia; 2 Department of Botany and Microbiology, Faculty of Science, Alexandria University, Alexandria, Egypt; 3 Department of Biological Sciences, Faculty of Science, King Abdulaziz University, Jeddah, Saudi Arabia

**Keywords:** sustainable agriculture, *Halocnemum strobilaceum*, arid land, biostimulant, eco-friendly salinity management, NaCl-induced abiotic stress, foliar application under salinity, salt stress mitigation in crops

In the **Abstract**, “This study assessed the effectiveness of *Halocnemum strobilaceum* extract (HE) as a foliar biostimulant to improve growth, yield, and physiological performance of *Chenopodium quinoa* (quinoa) under NaCl-induced salinity stress (0–150 mM).” has been corrected to read:

“This study evaluated the effectiveness of *Halocnemum strobilaceum* extract (HE) as a foliar biostimulant to improve growth, yield-related traits, and physiological performance of *Chenopodium quinoa* under controlled saline conditions (0–150 mM NaCl), representing productivity-limiting but non-severe salinity levels.”

In the **Abstract**, “HE enhances salinity stress tolerance by promoting osmotic adjustment“ has been corrected to read:

“HE application is associated with improvement in physiological traits and yield components by promoting osmotic adjustment”.

In the **Abstract**, “The SDS-PAGE results suggest de novo synthesis of stress related proteins, highlighting HE's role in modulating quinoa's proteomic response under high salinity.” has been corrected to read:

“The SDS-PAGE results show qualitative changes in the protein profile, highlighting HE's role in modulating quinoa's proteomic response to saline conditions.”

A correction has been made to the **Introduction**. “Therefore, the main aim of our study is to evaluate the potential cultivation of quinoa plants in saline soils, and to assess the ameliorative impact of the extract of *H. strobilaceum* plant as a bio-stimulant for salinity stress mitigation on quinoa plants” has been corrected to read:

“Therefore, the main aim of our study is to evaluate the potential cultivation of quinoa plants in saline soils and to assess the effect of *H. strobilaceum* extract as a bio-stimulant on growth and yield-related traits of quinoa under saline conditions”

A correction has been made to the **Introduction**. “The present study is innovative in exploring the potential of a naturally salt-tolerant halophyte as a sustainable source of bioactive compounds for enhancing salinity tolerance in quinoa, linking native halophyte resources with the improvement of stress resilience in cultivated crops.” has been corrected to read:

“The present study explores the potential of a naturally salt-tolerant halophyte as a sustainable source of bioactive compounds that may support quinoa performance under saline conditions.”

A correction has been made to the **Materials and methods** section. The following text has been added at the end of the subsection 2.2 Plant Materials, Growth Conditions and Treatment: “The experimental period extended from 17 November 2024 to 16 February 2025, corresponding to a total duration of 91 days (approximately 13 weeks). Salinity treatments was initiated 14 days after germination and maintained until the end of the experiment through regular irrigation with fixed NaCl concentrations; plant samples were collected after 8 weeks of sowing, while seeds were collected at the end of the experiment after 13 weeks. Plant samples were collected to analyze the following criteria.”

A correction has been made to the **Results** section. “To assess the effectiveness of *Halocnemum strobilaceum* extract (HE) in alleviating the detrimental effects of salinity stress on quinoa growth” has been corrected to read:

“To assess the effectiveness of *Halocnemum strobilaceum* extract (HE) in improving quinoa growth performance under saline conditions”

A correction has been made in the **Results** section. "Massive proline accumulation at 100 mM NaCl confirms its function as a key osmolyte under severe salt stress" has been corrected to read:

“Massive proline accumulation at 100 mM NaCl confirms its function as a key osmolyte under productivity-limiting saline conditions”.

A correction has been made to the **Results** section."The study indicated that some unique bands appear only with extract treatment at severe stress, indicating the de novo protein expression triggered by the extract." has been corrected to read:

“The study indicated that some unique bands appear only with extract treatment at 150 mM NaCl reflecting potential qualitative alterations in the protein profile that warrant future molecular validation.”

A correction has been made to the **Results** section. “Total protein was extracted from quinoa seeds and separated by SDS-PAGE. PAGE is a highly sensitive technique that makes it possible to identify micrograms of protein. It produces better separation of protein mixtures than other available techniques. These characteristics made this technique desirable for quantization and identification of proteins. Protein profiles revealed major differences among all treatments.” has been corrected to read:

“Total protein was extracted from quinoa seeds and separated by SDS-PAGE to visualize seed protein banding patterns among treatments. This electrophoretic technique enables the separation of complex protein mixtures based on molecular weight and allows the detection of proteins at microgram levels. The resulting profiles indicated observable variations in band intensity and presence/absence among treatments under saline conditions. However, the SDS-PAGE analysis presented here provides only a qualitative overview of protein pattern differences and does not allow precise identification or quantification of the individual proteins.”

A correction has been made to the **Discussion** section. The following text has been added after “Improving quinoa performance under salinity is therefore a key target for enhancing food security in regions facing soil and water salinization.”:

“It is important to emphasize that salinity tolerance in *Chenopodium quinoa* is strongly genotype-dependent with marked variations among cultivars and ecotypes in ion regulations, osmotic adjustment, photosynthetic stability, and yield responses (Ruiz-Carrasco et al., 2011; Bazile et al., 2016; Abdelazeem et al., 2025). Comparative studies of quinoa accessions from different Chilean regions have also demonstrated pronounced physiological and molecular variability under NaCl stress (Ruiz-Carrasco et al., 2011). The genetic variability of quinoa is huge, with cultivars of quinoa being adapted to growth from sea level to 4000 masl, from 40 degrees south to 2 degrees north latitude, and from cold, highland climate to subtropical conditions. This makes it possible to select, adapt, and breed cultivars from a wide range of environmental conditions (Jacobsen, 2003). The results of Cai and Gao (2020) further indicate that salinity tolerance in quinoa is mainly associated with efficient leaf osmoregulation, potassium ion retention and sodium ion exclusion, and overall ion homeostasis rather than enhanced antioxidative capacity. This framework aligns with foundational halophyte ecophysiology studies (Koyro et al., 2008) and established models of ionic homeostasis in quinoa, which highlight the critical role of Na^+^/H^+^ antiporters and osmotic adjustment (Shabala et al., 2013; Adolf et al., 2013). Accordingly, the K^+^/Na^+^ ratio in leaves or roots may serve as a reliable physiological marker for selecting and breeding salt-tolerant quinoa cultivars. Therefore physiological responses of *C. quinoa* can not be generalized at the species level, and our results obtained for the Miser-1 cultivar should be interpreted within this framework of genetic variability. Although quinoa is generally considered moderately salt-tolerante, its adaptive responses vary considerably within genotype and environmental conditions.”

A correction has been made to the **Discussion** section. The following text has been added after “K+/Na+ imbalance; while at high levels (100–150 mM), plants shifted toward hyperosmotic adaptation with elevated proline, amino acids, and Ca²+ accumulation to preserve membrane integrity.”:

“Notably, Fv/Fm values remained within the optimal range across salinity treatments, with a slightly higher value at 150 mM NaCl (0.7547) compared with the control (0.7276), indicating no severe photoinhibition of PSII. However, stable Fv/Fm does not imply the absence of salinity stress. In the quinoa cultivar Miser-1, PSII efficiency was maintained while growth and yield declined, suggesting that yield reduction under high salinity is mainly associated with morphological constraints, particularly reduced leaf area, rather than photochemical damage. This indicates a protective adjustment that preserves PSII function under stress at the expense of productivity.

A similar pattern has been reported in quinoa under other abiotic stresses. For example, Sven-Erik Jacobsen et al. (2005) showed that frost stress can markedly reduce quinoa productivity even when the photosynthetic apparatus remains relatively functional, indicating that yield losses may arise from stress-induced limitations on growth and development rather than direct damage to photosynthesis. Likewise, in the present study, salinity reduced productivity despite the maintenance of PSII efficiency, suggesting that yield decline is more closely associated with morphological and growth constraints than with photochemical impairment.”

A correction has been made to the **Discussion** section. “SDS-PAGE analysis revealed that salinity stress caused clear quantitative and qualitative changes in quinoa seed protein profiles.” has been corrected to read:

“The SDS-PAGE analysis provided a preliminary qualitative overview of quinoa seed protein patterns and suggested that saline conditions were associated with observable variations in band intensity and presence/absence among treatments.”

A correction has been made to the **Discussion** section. “Collectively indicating enhanced photosynthetic stability and reduced lipid peroxidation under severe but sub-lethal salinity." has been corrected to read:

“Collectively indicating enhanced photosynthetic stability and reduced lipid peroxidation under the applied saline conditions.”

A correction has been made to the **Discussion** section. “Overall, our findings demonstrate that *H. strobilaceum* extract (HE) mitigates salinity stress in *C. quinoa* by reducing oxidative damage, enhancing photosynthetic performance, and maintaining grain nutritional quality.” has been corrected to read:

“Overall, our findings demonstrate that *H. strobilaceum* extract (HE) is associated with improved physiological performance and maintenance of grain nutritional quality in *C. quinoa* grown under saline conditions.”

A correction has been made to the **Conclusion** section. “demonstrating its dual function in increasing stress resilience and maintaining grain nutritional quality. Collectively, these findings indicate that HE functions as a sustainable natural biostimulant capable of mitigating salinity-induced damage and supporting quinoa productivity under controlled conditions.” has been corrected to read:

“demonstrating its association with improved yield-related traits and the maintenance of grain nutritional quality. Collectively, these findings indicate that HE functions as a sustainable natural biostimulant capable of supporting quinoa productivity under productivity-limiting saline conditions.”

A correction has been made to the **Conclusion** section. “Foliar application of *Halocnemum strobilaceum* extract (HE) effectively enhanced quinoa (*Chenopodium quinoa*) tolerance to salinity by improving photosynthetic efficiency, reducing oxidative damage, stabilizing protein content, and sustaining yield traits even under high salt stress (150 mM NaCl).” has been corrected to read:

“Foliar application of *Halocnemum strobilaceum* extract (HE) was associated with improvements in several physiological and yield-related traits of quinoa under saline conditions.”

A correction has been made to the **References**. The following Reference details have been added:

“Abdelazeem Mousa, M., Veres, S., and Basal, O. (2025). Abiotic stress in quinoa: A comprehensive review on the impact of salinity and mitigation strategies. *Notulae Botanicae Horti Agrobotanici Cluj-Napoca*. 53(4), 14860. doi: 10.15835/nbha53414860.

Jacobsen, S.E., Monteros, C., Christiansen, J.L., Bravo, L.A., Corcuera, L.J., and Mujica, A. (2005). Plant responses of quinoa (*Chenopodium quinoa* Willd.) to frost at various phenological stages. *European Journal of Agronomy* 22 (2), 131-139. doi: 10.1016/j.eja.2004.01.003.

Adolf,V. I., Jacobsen,S., and Shabala,S. (2013). Salt tolerance mechanisms in quinoa (*Chenopodium quinoa* Willd.). *Environ. Exp. Bot*. 92, 43–54.

Bazile,D., Jacobsen,S., and Verniau,A. (2016). The Global Expansion of Quinoa: Trends and Limits. *Front. Plant Sci*. 7, 622.

Cai,Z. Q., and Gao,Q. (2020). Comparative physiological and biochemical mechanisms of salt tolerance in five contrasting highland quinoa cultivars. *BMC Plant Biol*. 20 (1), 70.

Jacobsen, S. E. (2003). The worldwide potential of quinoa (*Chenopodium quinoa* Willd.). *Food Rev. Int.* 19, 167–177. doi: 10.1081/FRI-120018883.

Koyro, H.-W., Geissler, N., Hussin, S., Debez, A., and Huchzermeyer, B. (2008). Strategies of Halophytes to Survive in a Salty Environment. In: *Abiotic Stress and Plant Responses*. pp. 83–104.

Ruiz-Carrasco, K., Antognoni, F., Coulibaly, A.K., Lizardi, S., Covarrubias, A., Martínez, E.A., Molina-Montenegro, M.A., Biondi, S., and Zurita-Silva, A. (2011). Variation in salinity tolerance of four lowland genotypes of quinoa (*Chenopodium quinoa* Willd.) as assessed by growth, physiological traits, and sodium transporter gene expression. *Plant Physiology and Biochemistry* 49 (11), 1333-1341. doi: 10.1016/j.plaphy.2011.08.005.”

Shabala,S., Hariadi,Y., and Jacobsen,S. (2013). Genotypic difference in salinity tolerance in quinoa is determined by differential control of xylem Na ion loading and stomatal density. *J. Plant Physiol*. 170 (10), 906–914.

The original version of this article has been updated.
